# BMI, a Performance Parameter for Speed Improvement

**DOI:** 10.1371/journal.pone.0090183

**Published:** 2014-02-25

**Authors:** Adrien Sedeaud, Andy Marc, Adrien Marck, Frédéric Dor, Julien Schipman, Maya Dorsey, Amal Haida, Geoffroy Berthelot, Jean-François Toussaint

**Affiliations:** 1 IRMES (Institut de Recherche bioMédicale et d'Epidémiologie du Sport), INSEP, Paris, France; 2 Université Paris-Descartes, Sorbonne Paris Cité, Paris, France; 3 Université de Rouen, CETAPS EA 3832, Mont Saint Aignan, France; 4 CIMS, Hôtel-Dieu, Assistance Publique des Hôpitaux de Paris, Paris, France; University of Sao Paulo, Brazil

## Abstract

The purpose of this study is to investigate the association between anthropometric characteristics and performance in all track and field running events and assess Body Mass Index (BMI) as a relevant performance indicator. Data of mass, height, BMI and speed were collected for the top 100 international men athletes in track events from 100 m to marathon for the 1996–2011 seasons, and analyzed by decile of performance. Speed is significantly associated with mass (r = 0.71) and BMI (r = 0.71) in world-class runners and moderately with height (r = 0.39). Athletes, on average were continuously lighter and smaller with distance increments. In track and field, speed continuously increases with BMI. In each event, performances are organized through physique gradients. «Lighter and smaller is better» in endurance events but «heavier and taller is better» for sprints. When performance increases, BMI variability progressively tightens, but it is always centered around a distance-specific optimum. Running speed is organized through biometric gradients, which both drives and are driven by performance optimization. The highest performance level is associated with narrower biometric intervals. Through BMI indicators, diversity is possible for sprints whereas for long distance events, there is a more restrictive aspect in terms of physique. BMI is a relevant indicator, which allows for a clear differentiation of athletes' capacities between each discipline and level of performance in the fields of human possibilities.

## Introduction

In general, nature tends to increase diversity and complexity [1]. In favorable circumstances this can be expressed by the expansion of phenotypic variability. Simple morphological traits, like mass and height of a species may follow this law. During the past century, populations of developed countries increased its stature, body mass, BMI and life expectancy [2]. Consequently, morphological diversity has increased in these populations. Diversity and complexity in sport encompass genetic, physiological capacities and psychological skills, in which morphological aptitudes equally play a role. Sport is considered as a selective system by virtue of its competitive nature [3]. One of the first selected characteristics is the physique. Consequently, recruiting morphological suitable athletes is common in most sports [4], [5]. Some studies demonstrated a link between morphology and success in track and field [6], [7]. BMI is an energy indicator relating total mass and height, which allows the comparison of athletes on various distances. In marathon runners, Marc et al [8] identified the most appropriate profiles and conditions to realize optimal performance. They found, at this time, that optimal BMI for men was 19.8 kg.m^−2^, and for the 10 best performers of all time a BMI range between 17.5 and 20.7 kg.m^−2^. Following the example of energy contribution from the aerobic to anaerobic mechanisms in different running distance [9], we hypothesized that phenotypic gradients exist among running events and levels of performance. The completeness of running events could reveal self-organization through biometric parameters, with coherence from sprints to long distance. The importance of physical traits in performance has not been thoroughly studied [10], as a result further studies are needed to enhance knowledge of running performance, including body mass, height and BMI as a performance determinant. Our purpose, is to study performance and anthropometric traits in order to reveal the strength of their association.

## Methods

### Data collection

Data of mass, height, BMI, and speed were collected for each international male athlete among the top 100 rankings of eight running events: 100 m, 200 m, 400 m, 800 m, 1500 m, 3000 m, 10.000 m and marathon during the 1996–2011 seasons. This represents 12,800 annual-performers and 3,852 different athletes. Height and mass values were coincided with each individual's best performance by year. All of the data was collected from the website http://www.tilastopaja.org and cross-classified by the International Association of Athletics Federations' site: http://www.iaaf.org.

### Data organization

Data was organized according to four types of distributions.

First, the distribution of all athletes by distance was organized according to their BMI to identify potential morphological gradients.

Secondly, by deciles of speed: the first decile represents the 160 best performers of the discipline and the last decile represents the 160 slowest performers for a total of 1,600 annual-performers by distance (Top 100 in 16 years). We compared data of mass, height and BMI according to race distances and performance deciles.

The third organization of data was by percentage of performance: we stratified athletes BMI, by distance treated by the percentage of the best performances during the study period (1996–2011).

Lastly, the fourth by density: distributions of all BMI points by running events were presented according to speed. In order to investigate these distributions, we partitioned the BMI points of all athletes according to running events depending on performance percentage over a mesh M. Let *X*, *Y* be the BMI of athletes and percentage of performance respectively, such that the data of an individual was expressed as *X_i_, Y_j_*, with 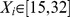 (*N*_X_  = 1600) and 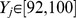 (*N*_Y_  = 1600). The density of athletes' BMI was estimated over the nodes of *M*. The boundaries of *M* were chosen in order to encapsulate all *X_i_* and *Y_j_*. Lower boundaries [Lx; Ly] were defined as the largest integer that does not exceed min(X_i_), min(Y_i_). Upper boundaries [Ux; Uy] were defined as the smallest integer that is not less than max(X_i_), max(Y_i_). Note that in our case, the difference of the boundaries of the athletes BMI dimension *X* was always greater than the one of percentage of performance *Y*:
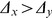
(1)


The numbers of nodes in the *X Y* dimensions were denoted *n_x_* and *n_y_* respectively, with respect to:
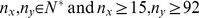
(2)


M was set as a homogeneous mesh, such that each node was separated by the value *a* in both dimensions, with:
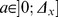
(3)


Such that the maximum possible distance between two nodes did not exceed *U_Y_* (1). The resolution *r* of *M* was given by:
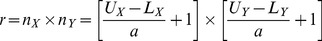
(4)


For the estimation of the density, the number of athletes' BMI performance *d_j_* falling into the area of a *j* (*j* = 1,...,*n*) node was summed, such that:
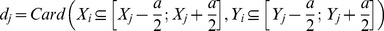
(5)is an estimate of the local density.

For choosing the best representation of the density and in order to avoid information loss due to an inadequate resolution of the mesh, we set the value of *a* = 0.8 as it was the most represented resolution among all distances. Additionally, we chose this specific representation because this mesh was the best for all track and field events.

### Statistical analysis

All of the data was reported as means ± standard deviation. Associations between the subjects' physique (height, mass and BMI), and speed were examined using the Pearson product-moment correlation coefficient. Differences in anthropometrics and speed of the different track and field running events were compared using one-way analysis of variance test (ANOVA). Comparisons of the different track and field groups were performed using Bonferroni's Multiple Comparison Test. The level of significance was set at p = 0.05. Statistical analyzes were realized with the software Statistica 7.1 and Matlab 7.13.

### Ethics

This study is designed and monitored by the IRMES (Institut de Recherche bio-Médicale et d'Epidémiologie du Sport) scientific committee. It uses a research protocol qualified as non-interventional, in which ‘…all acts are performed in a normal manner, without any supplemental or unusual procedure of diagnosis or monitoring.’ (Article L1121–1 of the French Public Health Code). According to the law, its approval therefore did not fall under the responsibility of a committee for the protection of persons (CPP), it does not require informed consent from individual athletes.

## Results

Speed is significantly associated with mass (r = 0.71) and BMI (r = 0.71) but moderately with height (r = 0.39). The ANOVA test shows significant differences among events for height (excluding 10,000 m *vs* marathon, 200 m vs 800 m), mass (excluding 10,000 m *vs* marathon and 100 m *vs* 200 m and 400 m, and 200 m vs 400 m) and BMI (excluding 3,000 m *vs* marathon).

### Mass, height and performance

**Figure 1:**
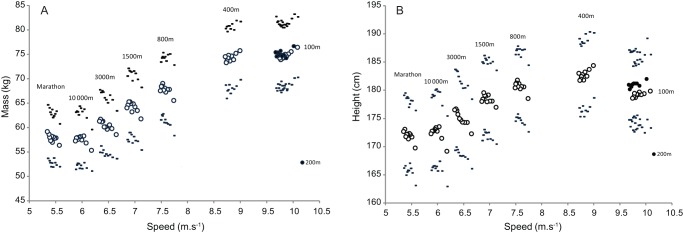
Mean mass (A) and height (B) ± SD of athletes in each decile of speed for the eight events (100m, 200m, 400m, 800m, 1500m, 3000m 10 000m and marathon). Black circles show the 200

The mean mass of athletes by decile and discipline continuously increases with speed (Figure 1A). For short distances (100 m, 200 m and 400 m) athletes are heavier (74.82±6.39 kg 75.10±6.61 and 74.38±6.38 kg respectively) than others runners. When distance increases, mean mass of the runners decrease (800 m: 67.78±6.5 kg, 1500 m: 64.19±6.58 kg, 3000 m: 60.45±5.79 kg, 10,000 m: 57.49±5.46 kg; marathon: 57.85±5.11 kg).

The mean height of athletes by decile and discipline also depends on speed (Figure 1B). Smaller athletes run long and middle distances, with a progressive increase in mean height from marathon to sprint events (marathon: 171.9±6.28 cm, 10,000 m: 172.37±6.44 cm, 3000 m: 175.02±6.55 cm, 400 m: 182.75±6.24 cm, 200 m:180.99±6.17 cm and 100 m: 179.20±5.94 cm).

### Gradients with level of performance

First decile athletes from marathon to 800 m are lighter than their counterparts in lower deciles. Conversely, a break occurs in sprints (400 m, 200 m and 100 m), where the most successful athletes (from the first decile) have a gradient tending towards a higher mass. Thus, the fastest athletes in sprints are heavier while the lighter athletes are the most effective in long distances. Like mass, athletes of the first deciles from marathon to 800 m are shorter than their counterparts in the lowest deciles. In contrast for sprints, a break occurs as well for the most successful athletes who they display a progressively taller height.

### BMI: gradients of physiques

**Figure 2:**
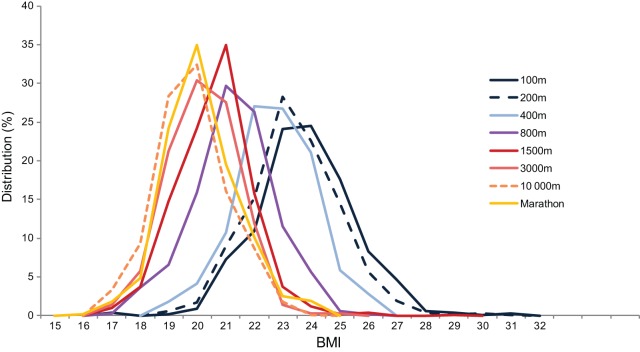
BMI distribution of all athletes by running events. Each curve links points representing the percentage of athletes per 1 BMI unit for each event.

Figure 2 shows the BMI distribution of all athletes by running events. The highest percentage of athletes is seen at 24 kg.m^−2^ for the 100 m, 23 kg.m^−2^ for the 200 m, 23–22 kg.m^−2^ for the 400 m, 21 kg.m^−2^ for 800 m and 1500 m and 20 kg.m^−2^ for the 3000 m, 10 000 m and marathon. Long distances are distributed according to a peak while, 100 m 400 m have a plateau with range of BMI.

### BMI and performance

**Figure 3:**
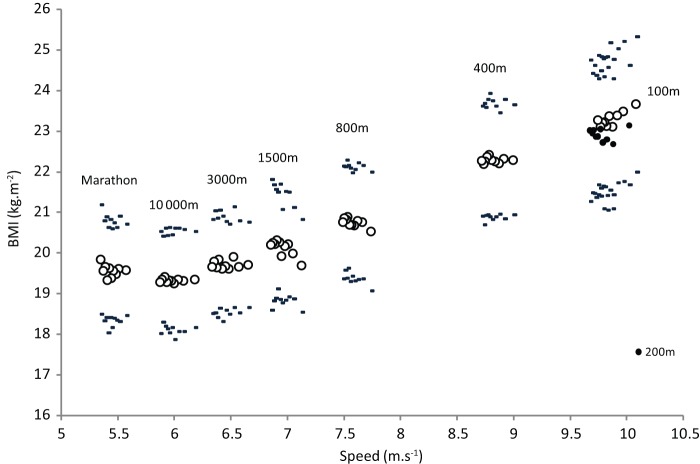
Mean speeds and SD according to BMI for each speed decile and each events. Black circles show the 200 m athletes ordered by decile.

Figure 3 shows the mean BMI of athletes by decile and by discipline, according to speed. There is a continual increase in BMI with speed improvement from marathon: 19.57±1.29 kg.m^−2^ to 100 m: 23.3±1.67 kg.m^−2^.

**Figure 4:**
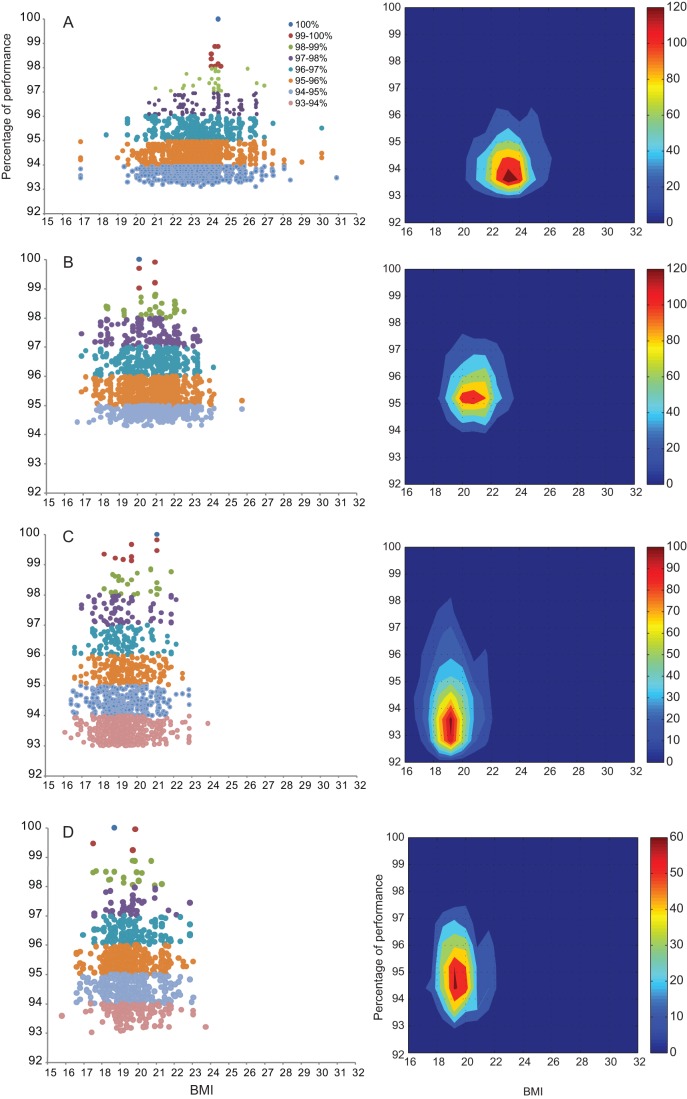
BMI of all athletes according to their performance. To the left: Exact data of athletes' BMI distribution. To the right, athletes' BMI are represented by a density function. At the left end points are more visible to the right central density of greater number of athletes appears more clearly. (Fig. 4A 100 m, Fig. 4B 800 m, Fig. 4C 10 000 m and Fig. 4D marathon).

Exact data and density function are shown in Figures 4 ordered by distance and classified by percentage of performance (100%  =  best performance during the period studied) and function of BMI. For the four distances, the greater the level the more tightened the BMI spectrum. For example in the 800 m, when performance level reaches 93–94% of the best one, BMI ranges from 16.7 to 25.7 kg.m^−2^; when performance level reaches 98–99%, BMI ranges from 20.1 to 20.9 kg.m^−2^. And we observed the same differences across all of the events. We also observed an offset of the majority of the points (red density) towards lower BMI from sprint events to long and middle distance. For the 10,000 m and marathon, like the best performers, the greatest numbers of points (red density) are centered on an optimum interval between 19–20 kg.m^−2^.

## Discussion

The present study shows that biometric parameters are ordered in a consistent self-organization between sprint and long distance. Physique optimal range for performance across the full continuum of event specializations events emerge in an organized structural basis. Consequently, this study is the first to reveal morphological optimization on the entire spectrum of track events and the relevance of BMI as performance indicator.

We also find that between 1996 to 2011 seasons, mean mass and height of the best athletes of sprint events (100 m to 400 m) are bigger (BMI and mass) than those of middle and long distance (800 m to marathon). This confirms the trends observed in track and field history [11]–[13] and are consistent with more recent studies [6], [10], [14].

### Different height for different events

As distance progressively decreases from marathon to 400 m, the runners gradually become taller, in accordance with the literature [15], [16]. This trend is not continuous, 100 m and 200 m athletes are on average shorter than those of the 400 m. The fact that 400 m athletes are the tallest, is in accordance with other studies [6], [14], [17], [18]. Being taller in this distance may confer benefits like improvements in stride length [19]. Locomotion is a dual time organization: vertical loss of useful energy (lifting the body mass, which later drops), and the horizontal loss caused by friction against the surrounding medium [20]. For taller athletes, mass that falls from a higher altitude falls faster, down and forward [21]. Bejan and Marden [20] also show that the speed-height relation is predictable from the power law applied to animal locomotion. Speed increases with larger physiques in different species including mammals and human. For example, 3 percent increase in the height of the center of mass means a 1.5 percent increase in runners speed [21]. The fact that 100 m runners are shorter than their 400 m counterparts highlights another hypothesis. First, like O'Connor et al [6] suggest, longer legs reduce stride rate. Moreover relatively shorter thighs diminish the resistance leverage on the upper leg and the cost of locomotion. Second, in short sprint events, start, reaction time and the acceleration phase are crucial. Smaller runners own better reaction time [22]. For the movement, the response time depends on the length of the body especially the lower limbs and muscle fasciles lengths [23] consequently shorter athletes draw benefits in starting blocks. Furthermore, shorter legs will generally have a lower moment of inertia, and hence require less energy to accelerate [6]. A range of these characteristics could explain why 100 m sprinters are smaller than the 400 m ones.

### Mass requirements and performance

Similarly to height, as the distance progressively decreases from marathon to 100 m, runners gradually become heavier. This redefines mass as a key requirement for speed [24]. These results are consistent with the constructal theory of Bejan and Marden [20], which states that speed increases with mass. Moreover, heavier body mass is associated with improved efficiency in sprint events, due to the necessity of muscle strength, ground force and power [14] and to improved return of elastic energy via the stretch shortening cycle [25]. On the other side of the distance spectrum, smaller stature and mass could provide an advantage for marathon runners. The metabolic cost of horizontal forward motion will in principle increase with mass [6], the heavier the athletes, the lower his energy cost [26], [27]. Indeed, body mass is a significant determinant of running economy [28] and could generate poor mechanical efficiency [29]. Some authors [30], [31] hypothesized that the superior running economy of the Kenyan runners is primarily due to their slender limbs with lower masses requiring less muscular effort in leg swing. Another factor is that ground reaction forces are reduced in lighter runners than heavier ones [10]. In order to maintain the high mileage and high intensity load sustained during training, athletes of limited mass have an advantage by an attenuating shock effect [10]. Pugh [32] supports that a larger body size increases the air resistance to running, and consequently smaller and leaner runners will also a lower air drag during running. Gravity is the major force to overcome during running: in this case, excess mass is detrimental to running performance [33]. An excess of body mass, means that greater muscular effort and higher energy expenditure is required [34].

Smaller sized runners also draw another benefit of their morphologies for long distances. There is a strong relationship between distance and heat-exchange characteristics. Heat production/dissipation ratio becomes increasingly important as running distance increase [6]. Body mass increases thermal strain, heavier runners reach a heat storage limit sooner than lighters ones [35]. The apparent thermodynamic advantage of lighter runners may allow them to run more intensely or longer before reaching a limiting core temperature [10], [35], [36]. This could be advantageous not only in competition but also in training, especially at high intensity [10].

### Reinforced link with performance increases

Links between performance and morphology are strengthened by gradient of size within each discipline. Not only sprinters are heavier than their long and middle distances counterparts but within their distance, the fastest athletes are also heavier. This confirms the trend observed by Khosla [11], [12]: olympic champions in sprinting events are heavier than the finalists and the other participants. In contrast, the best long distance runners have a lower mass compared to less rapid athletes, like gold medalists versus finalists and other participants during Munich and Montreal Olympics games [12]. We find similar trends regarding height; the most successful sprint runners have a greater average height, whereas the best performing endurance runners have a smaller height than their slower opponents. Our study shows, through physique gradients, the importance of mass and height in all track and field events.

### BMI, performance and optimal range

Like energetic progressive contribution from the aerobic to anaerobic mechanisms [9], BMI gradients exist with distance increments. Indeed, biometric parameters are ordered and show a consistent self-organization between long distance and sprints. While BMI is a useful indicator in the general population as a public health indicator, it appears that it is also a relevant indicator in order to differentiate athletes between each discipline.

A consistent trend of increasing BMI with speed was observed with distance running performance, in accordance with previous studies showing positive effect between BMI and performance [29], [37]. Elite sprinters are heavier due to their need of higher energy outputs in a short amount of time. This corresponds to a maximization of anaerobic metabolism, mainly involved in the total energy requirements in 100 m [9]. Indeed, ATP concentration is dependent on muscle mass, and BMI among athletes represents an indication of the power reserve related to lean mass [38], [39]. Possible progression in speed resides in mass increments [14].

Event differences, from the marathon to 100 m, create patterns of divergent BMI and optimal body type. As performance increases it can be observed that the spectrum of BMI narrows into a more optimal area. Our study shows a reduction in variability of BMI with performance increments, where the best athletes are attracted to optimum interval. For an athlete, being away from optimum probably negatively affects his performance. Moreover, a major part of the 10,000 m and marathon athletes are also centered around an optimum interval (19–20 kg.m^−2^) respectively, as shown by Marc et al [40]. This evidence could indicate that there may be an optimal structure of body suited to marathon and other long distance races [35]. Despite centering on an optimum attractor for all distances, certain diversity is possible for sprints up to 800 m while, density functions show a much smaller range of possibilities for 10,000 m and marathon races. This could be explained by the more restrictive aspect in terms of physique on athletes during long distances, a phenomenon enhanced by selection. The narrowing range with performance could relate the best biomechanical (e.g.torque-angle and force-velocity relationships), bio-physical (surface area-to-mass ratios) and physiological (energy release) adaptive components on each distance [4], and express the combination of ideal attributes [41]. It may illustrate the best trade-off by distance between different requirements in multi-objective optimization problems [42]. This could explain the decreases of BMI when race distance increases. This could also represent a functional trade-off between mass-specific aerobic power and endurance *vs* additional musculoskeletal structure required to run faster [14]. Additionally; this illustrates the energy needs during different races, assessing by an indicator of the embedded energy: BMI.

### The base of a pattern between and within different species

Locomotion is one of the major functions in life. Previous studies have suggested that the relationship between maximum relative running speed and body mass follow a curvilinear function [43] and indicated that a similar pattern may be found between mammal species. For example, species used in racing competition (*Canis Lupus*, *Equus Caballus*) co-evolved with humans and share a common training pressure [44]. In fact there is a common pattern apply for racing greyhound, including mass optimal area for best performance and centration around a mass optimum interval (personal data). Moreover, it will be very interesting to investigate BMI as indicator of embedded energy and BMI/exercise phenotype pattern in different mammals included quadrupeds.

## Conclusion

This study emphasizes mass, height and BMI as key requirements for speed. It allows for the identification of optimal physiques according to track and field events. BMI and mass are better indicators than height. However, BMI is preferred because it allows for the combination of both contributions. It appears to be a useful indicator in the categorization of elite athletes. Over time, physiological, physical and biomechanical constraints generated morphologies adapted to each race, a trend reinforced by performance gradients within each discipline. As a result there is a narrowing range around an optimal BMI for each event, where best athletes are “attracted”. Our study also reveals a possibility of larger organization induced by BMI range in diversity and complexity increase system.
